# Three-Dimensional Bioprinting Nanotechnologies towards Clinical Application of Stem Cells and Their Secretome in Salivary Gland Regeneration

**DOI:** 10.1155/2016/7564689

**Published:** 2016-12-20

**Authors:** Joao N. Ferreira, Sasitorn Rungarunlert, Ganokon Urkasemsin, Christabella Adine, Glauco R. Souza

**Affiliations:** ^1^Department of Oral & Maxillofacial Surgery, Faculty of Dentistry, National University of Singapore, Singapore; ^2^National Institute of Dental and Craniofacial Research, National Institutes of Health, Bethesda, MD, USA; ^3^Department of Preclinical and Applied Animal Science, Faculty of Veterinary Science, Mahidol University, Nakhon Pathom, Thailand; ^4^The University of Texas Health Science Center at Houston, Houston, TX, USA; ^5^Nano3D Biosciences (n3D), Houston, TX, USA

## Abstract

Salivary gland (SG) functional damage and severe dry mouth (or xerostomia) are commonly observed in a wide range of medical conditions from autoimmune to metabolic disorders as well as after radiotherapy to treat specific head and neck cancers. No effective therapy has been developed to completely restore the SG functional damage on the long-term and reverse the poor quality of life of xerostomia patients. Cell- and secretome-based strategies are currently being tested in vitro and in vivo for the repair and/or regeneration of the damaged SG using (1) epithelial SG stem/progenitor cells from salispheres or explant cultures as well as (2) nonepithelial stem cell types and/or their bioactive secretome. These strategies will be the focus of our review. Herein, innovative 3D bioprinting nanotechnologies for the generation of organotypic cultures and SG organoids/mini-glands will also be discussed. These bioprinting technologies will allow researchers to analyze the secretome components and extracellular matrix production, as well as their biofunctional effects in 3D mini-glands ex vivo. Improving our understanding of the SG secretome is critical to develop effective secretome-based therapies towards the regeneration and/or repair of all SG compartments for proper restoration of saliva secretion and flow into the oral cavity.

## 1. Introduction

Irreversible salivary gland (SG) damage and dry mouth (or xerostomia) are commonly present in a vast range of systemic conditions (e.g., Sjögren's syndrome, uncontrolled diabetes, and thyroid disease), and it is particularly severe after radiotherapy (RT) for head and neck cancers (HNC) [1]. On an annual basis, about 500,000 new cases of HNC develop worldwide for whom xerostomia-induced RT is the main treatment modality. Saliva secretions are essential for digestion, lubrication, oral homeostasis, and protection against a variety of environmental hazards. Hence, xerostomia can cause various life disrupting side effects such as oral infections, pain, and tooth loss. These side effects will impair daily activities related to taste perception, speech, mastication, and swallowing [2]. Salivary secretion has partial improvements after novel modalities, such as SG sparing or intensity-modulated radiation therapy, are utilized [2–4]. Despite these recent efforts, about 40% of dry mouth cases are still irreversible. When the radiation field (during RT) lays on the SG, radiation damage is elicited on the secretory epithelial cell compartment, blood vessels, and adjacent nerves [5, 6]. Following RT, patients lose the majority of acinar epithelial cells (about 80% of total epithelial cells) with the surviving secretory cells being primarily ductal; consequently, RT will irreversibly impact salivary secretion and cause inflammatory damage and fibrosis on the long-term. This radiation damage further depletes the SG stem/progenitor cell niche deterring healing and natural gland regeneration [5, 7–9]. Yet, no effective therapy has been devised to treat RT-induced xerostomia, and current treatment strategies are confined to the minimization of SG radiation damage or to the administration of artificial saliva substitutes and stimulators of saliva secretion (e.g., pilocarpine) [2, 5].

Radiation-induced xerostomia can be an irreversible life-long condition that can significantly affect the quality of life of HNC patients. Thus, novel and effective therapeutical strategies for SG hypofunction are required [10]. Due to the depletion of the self-renewable progenitor/stem cell pool during RT damage, cell-based therapies are essential not only to generate new saliva-secreting tissues [10–13] but also to potentially repair the damaged SG via the production and extracellular release of bioactive secretory proteins by transplanted cells [14–17]. This group of non-membrane-bound secretory proteins has been named the salivary secretome [18]. According to the human secretome atlas, salivary glands produce the most abundant proteins found in the human body [18]. Important cellular differences exist within the three major salivary glands (parotid, submandibular, and sublingual), mostly in the ratio of serous to mucous epithelial acinar cells and potentially in their pool of progenitor/stem cells. Despite these differences, researchers mainly focused their secretome-based and SG regenerative studies with 3D systems on either the submandibular or the parotid glands. The salivary secretome produced by different stem/progenitor cells will be discussed in the next sections since it could transform the way we restore the salivary flow in patients with xerostomia in the near future.

## 2. Salivary Stem/Progenitor Cells and Their Secretome

The first proof of concept study on transplantation of autologous SG cells to rescue salivary hypofunction using in vitro floating spheroid-like cultures of mouse SG progenitor cells, named salispheres. In vitro salisphere cultures have been shown to enrich SG stem/progenitor cell populations that include KIT (C-KIT, CD117), Sca-1, and Mushashi-1 [11]. KIT-expressing (KIT+) progenitors are also found in other epithelial organs beside the SG, such as the prostate gland and lungs, where KIT+ progenitors have remarkable regeneration capabilities [20, 21]. In a salisphere study in mice, 100–300 KIT+ donor-derived cells isolated from the salisphere cultures were sufficient to form both new acini and saliva-transporting ductal structures, restoring the morphology and function of irradiated SG. Since human salispheres do contain KIT+ cells, there is a potential for future clinical use of KIT+ cell subpopulations [22]. Recently, Pringle and others [13] have successfully transplanted human salispheres into irradiated mice restoring the salivary flow, particularly when these salispheres were positively selected for KIT. However, the subpopulation of KIT+ cells in human SGs is very limited being less than 0.4% of the total population in younger adults, and this number substantially decreases with aging [13]. Moreover, these salispheres have a restricted in vitro self-renewal and proliferative capacities that confines their growth to 2-3 population doublings at earlier passages (P1–P4) [13].

Thus, it is crucial to understand how progenitors proliferate and expand particularly during organogenesis. Several researcher groups have demonstrated that KIT and fibroblast growth factor receptor 2b (FGFR2b) signaling are essential for progenitor survival and expansion in the fetal submandibular gland, lung, pancreas, tooth, and skin [39–41]. Moreover, other putative markers can be used to isolate SG stem/progenitor cells including KRT5 (Cytokeratin 5), CD49f, CD29 (Itga1), CD133 (Prom1), Sca1, CD44, CD34, CD90 (Thy1), CD105, CD9, and CD81, but only few populations were proven to actively restore damaged glands [11, 42–45]. Yet, the KIT+ cell population still appears to have the highest stem/progenitor-like potential.

Research efforts have been made to increase the number of KIT+ cells ex vivo using growth factors [32] or to administer secretome factors to reverse SG damage in vivo [60]. Several secretome components have been studied including specific heparan sulfate peptides [32] and several growth factors and cytokines (see Table 1 for a complete list). The majority of these secretome components (EGF, IGF1, FGF2 [26, 27, 33], FGF7 (or KGF) [28], IL-6 [34], ALDH3 [24], or EDA activators [25]) have similar cellular downstream effects such as the reduction in cell apoptosis and/or the promotion of epithelial proliferation. These secretome-based strategies could be advantageous, although the absolute cell number required for functional regeneration of the human SG is still unknown. Instead, non-SG cells may be considered to curb this constraint.

Taken together, multiple research groups have shown that rodent SG-specific epithelial cell transplantation is a feasible approach to repair irradiated SGs. Future studies will determine whether human SG cells behave in a similar manner in ex vivo and in vivo assays [13]. While success has been achieved with epithelial KIT+ cells in rodents, currently, other more multipotent stem/progenitor cell candidates and/or compartment reservoir cells can be investigated (e.g., cytokeratin 14) [61]. Despite this, in clinical scenarios where autologous SG cell numbers are reduced, we may need to take advantage of the regenerative capacity of non-SG stem cells, nonepithelial cells (e.g., bone marrow-derived), or simply their secretome. These potential therapeutical options are reviewed in the following section.

## 3. Nonsalivary Gland Cells and Their Secretome

There are a vast number of reports on the advantageous effects of non-SG stem cells and their secretome to regenerate irradiated SGs (see Tables 2 and 3). These reports include several types of stem cells such as bone marrow- (BM-) derived cells [63, 64], BM-derived mesenchymal stem cells (MSCs) [14, 52], human adipose-derived MSCs [53, 54], SG-derived MSC-like cells [55], amniotic cells [56, 57], embryonic stem cells (ESC) [58], and induced-pluripotent stem cells (iPSC) [59].

Recently, BM-derived transplants using either mesenchymal stem cells (MSC) or BM secretome (also named “soup” or “bioactive lysates”) have been shown to induce paracrine prosurvival effects on remaining SG tissues towards a more functional SG tissue architecture [14, 15]. When intraglandular transplantation of BM cells and their secretome was implemented, the outcomes in irradiated mouse SG were promising; and those included an improvement in saliva production, reduction in apoptosis, and changes in microvessel density [15]. Earlier studies in mouse irradiated SG had similar functional outcomes, when BM-derived cells were mobilized by G-CSF/FLT3/SCF [50, 62]. The clinical translation of these cellular paracrine effects led investigators to identify such bioactive secretome components secreted by BM-derived cells [15, 16]. Protein microarrays detected several angiogenesis-related factors (CD26, FGF1, HGF, MMP-8, MMP-9, OPN, PF4, and SDF-1) and cytokines (IL-1ra, IL-16) in the BM secretome (Table 3) [16]; thereby, several signaling pathways may be involved and the contribution of each secretome component towards epithelial repair and SG regeneration requires further investigation.

Despite tentative differentiation of BM-derived cells and MSCs into SG acinar cells in vitro, their actual contribution to epithelial differentiation in vitro and in vivo is puzzling. Highly homogenous BM clonal MSC (BM-cMSC) has recently shown potential to regenerate SGs, although the current mechanisms of regeneration are not well understood [14]. In addition, an in vitro study using BM stem cells (BMSCs) cocultured with neonatal rat parotid acinar cells showed an increase in the induction of acinar-specific *α*-amylase expression in BMSCs [51]. This coculture scenario with mesenchymal and epithelial stem/progenitor cells can be an interesting therapeutical approach when used in combination with relevant secretome factors. Further studies are still needed to test the secretory function of these acinar-like cells from bone marrow sources. As somewhat expected, both BM-MSC and mesenchymal-like cells derived from SG can suppress the immune system [65].

Interestingly, researchers have also looked at adipose sources of stem cells. Human adipose-derived mesenchymal stem cells (hAdMSCs) via systemic administration exhibit improved salivary flow rates 4 months after radiation therapy [54]. Glands with hAdMSC transplants showed lesser epithelial acinar apoptosis and tissue fibrosis and higher secretory mucin and amylase levels. At 4 weeks, a large number of infused hAdMSCs were detected in vivo and were found to have differentiated [54]. Moreover, the secretome from hypoxia-preconditioned hAdMSC comprised high levels of GM-CSF, VEGF, IL-6, and IGF-1 (Table 3) [17]. This hAdMSC secretome strongly induced epithelial proliferation and exerted antiapoptotic effects in the SG in vivo. A common finding across these adult stem cell secretome studies is the presence of secretome-based paracrine effects to reduce radiation-induced epithelial apoptosis, proliferate the host SG progenitor cells, and induce angiogenesis.

The known components of the secretome derived from adult stem cells are summarized in Table 3 since they are multiple. The antiapoptotic, proproliferative, and proangiogenesis cues found in the secretome can support not only the repair of the epithelial cells but also the microenvironment [17]. However, the following question can be posed: could the secretome strategy be a successful therapy in every patient, particularly for the patients without any remaining SG cells left after radiotherapy? The secretome strategy like the current ones involving salivary stimulation (e.g., stimulation with oral pilocarpine tablets) relies on the amount of remaining SG cells; thus, clinical outcomes will depend on the remaining cells that need paracrine stimulation.

While proangiogenesis factors have been reported in certain secretomes, it is not known yet whether neurotrophic factors are present [66]. Parasympathetic neurons are known to support epithelial regeneration after RT [43, 60]. Neurotrophic factors such as neurturin (NRTN) or glial cell-derived neurotrophic factor (GDNF) are currently being tested to revert the hypofunctional status of irradiated SGs [43, 60].

Other pluripotent cell types such as ESC and iPSC have recently been investigated as new cell sources to generate mature salivary gland cells [58, 59]. A study with mouse ESCs cocultured with human SG-derived fibroblast has provided (to ESCs) the cues to express SG-specific markers and to reconstitute SG structures; however, it is still unclear whether SG function can be restored [58]. Both ESC- [58] and iPS-derived SG cells [59] have the potential to be an adjuvant cell-based therapy as long as properties such as genomic stability and lack of tumorigenesis are secured at transplantation.

Nonetheless, in clinical scenarios where whole new SG organs or mini-glands are necessary for in vivo transplantation, three-dimensional (3D) SG in vitro culture systems (with or without bioscaffolds) are required to integrate multiple cell lines (under specific growth factor conditions) for the generation of all gland compartments (acinar and ductal epithelial, myoepithelial, endothelial/vascular, and neuronal).

## 4. Generating Salivary Gland Organoids/Organs and the Role of 3D Bioprinting

A recent breakthrough in the field of SG whole organ regeneration showed that a bioengineered gland made from fetal epithelium and mesenchyme can be transplanted into an adult mouse to form a new whole functional gland in the adult microenvironment [67]. This bioengineered gland contained a variety of embryonic cells, including progenitors of epithelial, mesenchymal, endothelial, and neuronal cells. Importantly, the gland reconnected with the existing ductal system and was functional in terms of saliva secretion, protection of the oral cavity from bacteria, and restoration of normal swallowing. Thus, this concept may lead to the creation of new surgical techniques for the prompt implantation of ex vivo SG organs to integrate with the existing circulatory and nervous system structures and align endogenous salivary ductal structures. However, this mouse model system may not fully translate into clinics due to the use of fetal glands. Thus, this major advance prompted researchers to develop 3D organotypic cultures to produce SG organoids or mini-glands that can recapitulate the in vivo native environment and SG morphology and architecture [10].

**Figure 1:**
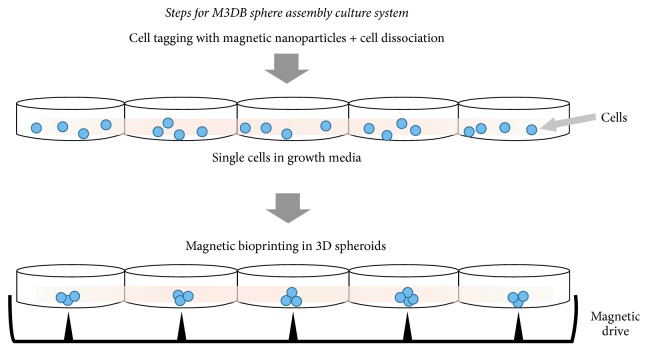
Diagram showing magnetic 3D bioprinting (M3DB) sphere assembly culture system by magnetic force driven patterning of tagged cells [19].

**Figure 2:**
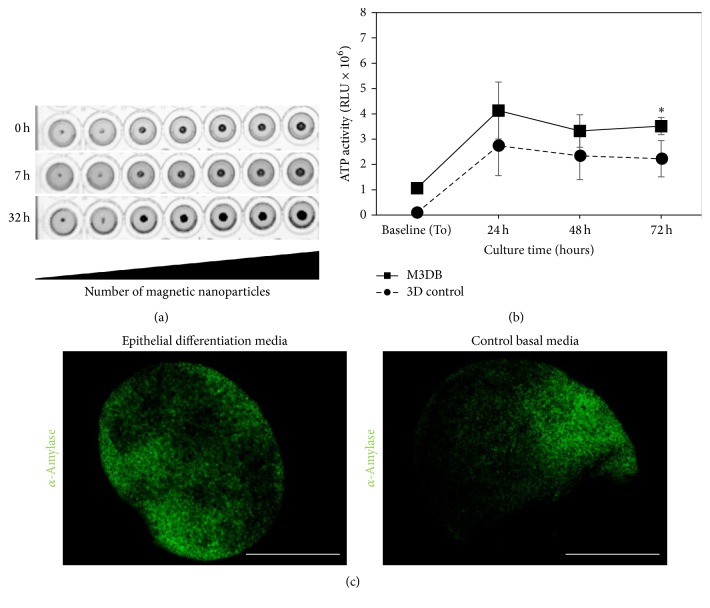
Morphology and viability of the M3DB spheroid-like organoids after 3D bioprinting of human dental pulp stem cell (hDPSC) cultures in a 96-well plate. (a) Morphology of the M3DB spheroids after 7 h and 32 h of culture of 3 × 10^5^ hDPSC using increased concentration of magnetic nanoparticles for cellular tagging and magnetization. (b) ATP activity of M3DB compared to a conventional 3D system (3D control) from baseline to 72 hours after seeding 1 × 10^5^ hDPSC at baseline (time 0 h). ATP activity was measured by a luciferase ATP-based 3D assay (CellTiter-Glo 3D Cell Viability Assay, Promega, USA) with a Glomax luminometer (RLU: raw luminescent units); significant difference found between the two culture systems (M3DB and 3D control) at 72 h (^*∗*^ *p* = 0.0286); *N* = 4-5; Two-tailed* t*-test. (c) Organoids expressing *α*-amylase salivary protein after epithelial differentiation (GlutaMAX basal media with FGF-10 40 ng, Gibco) of hDPSC for 14 days. Organoids were processed for whole mount immunofluorescence staining with *α*-amylase primary antibody and Alexa Fluor® 488 (green) followed by confocal fluorescence microscopy. Images are a maximum intensity projection of a z-stack of images taken through the entire organoid thickness (magnification: 10x; scale bar: 250 *μ*m).

As a result, novel 3D bioprinting nanotechnologies have been recently developed using magnetic patterning or levitation, in which cells bind with a magnetic nanoparticle assembly overnight to render them magnetic [19]. These bioprinting systems are time efficient as they require less than 24 hours of working time to assemble cells in 3D, depending on the cell type and number of magnetic nanoparticles used (Figures 1 and 2(a)) [68, 69]. Their magnetic nanoparticle assembly includes gold, iron oxide, and poly-L-lysine, which can easily tag different cell types at the plasma membrane level. When resuspended in medium, an external magnetic field levitates and can concentrate different SG cells at the air-liquid interface, where they aggregate to form larger 3D organoids (Figures 1 and 2(a)). The resulting dense cultures can synthesize extracellular matrix and can be analyzed similarly to other 2D/3D culture systems, using assays/techniques such as cytotoxicity assays, immunohistochemical analysis, western blotting, and other biochemical assays [70]. These 3D bioprinted systems have been previously found to recapitulate the native extracellular matrix from several tissues such as fat, lung, aortic valve, blood vessels, and breast and glioblastoma tumors [19, 68, 69, 71–74].

These magnetic-based bioprinting strategies are an avenue that we are currently exploring since their biocompatibility is comparable to conventional 3D systems using centrifugation-based force aggregation (Figure 2(b)). These bioprinting cell assembly systems can integrate all human SG cellular compartments (acinar/ductal epithelial, myoepithelial, endothelial, and neuronal) into organotypic cultures. More interestingly, these 3D bioprinting systems have been tested in cultures with oral stem cells such as human dental pulp stem cells (hDPSC) in combination with secretome components (e.g. FGF-10) and have shown to produce *α*-amylase-secreting cells (Figure 2(c)). However, the polarity in these secretory epithelial cells still needs to be evaluated.

During the development of the SG organoid, the creation of the apicobasal polarity in epithelial cells and of branched lumenized ducts is paramount to achieve a proper directionality for the salivary flow and production of saliva. These epithelial polarity properties of the SG organoids or mini-glands have been difficult to achieve [75]. However, these bioprinting strategies have shown promise when applied in in vivo rodent models using magnets [76]. In this particular in vivo study, the magnetized stem cells were biocompatible and successfully targeted a locally damaged neuronal tissue restoring its function.

Taken together, these innovative magnetic-based 3D bioprinting strategies are relevant in the SG regeneration field because they may (1) first generate scaled-up xeno-free biocompatible 3D tissue compartments that provide an architecture with environmental cues to support cell growth, differentiation, and biointegration in the remaining tissues (after damage) to restore homeostasis and functionality; (2) secondly they may establish coculture methods to generate SG cell-derived secretome, matrices, and tissue compartments on a scaled-up manner. These cocultures will allow researchers to integrate, in a 3D architecture, the complexity of different human SG component; and (3) lastly test new surgical techniques using magnetic fields in vivo to promptly implant and hold/stabilize magnetized SG organoids/mini-glands onto the injury site [76].

## 5. Future Directions

There has been a research trend towards the development of secretome-based therapeutical strategies to repair and/or restore salivary glands (SG) damaged by radiotherapy. These strategies have been relatively successful in rodent models for the clinical scenarios where the majority of SG cells and tissue compartments still remain. Nonetheless, when a patient needs a whole new SG, organotypic 3D cell culture systems are required to generate robust 3D organoids or mini-glands ex vivo for proper acinar epithelial stimulation, saliva production, and release into the oral cavity. These 3D mini-glands can be established using coculture systems to integrate in 3D the complexity of the different SG cellular/tissue components, such as epithelial acinar and ductal cells, myoepithelial cells, the networks of parasympathetic nerves, and lumenized ducts and vessels. For this purpose, novel 3D bioprinting approaches have been developed to assemble all the above SG cells in coculture and produce 3D tissue compartments and ductal structures that resemble mini-SG.

In summary, secretome-based and 3D organotypic cell-based strategies will certainly become the next generation of biomedical therapies to either repair a damaged SG or to develop an in vitro SG organoid/mini-gland for transplantation in humans suffering from xerostomia.

## Figures and Tables

**Table 1:** List of secretome components (matrix peptides, cytokines, growth factors, and enzymes) from SG cell lines that can be potentially used in SG regeneration strategies. More details about each secretome component can be found in [18, 23]. ALDH3: aldehyde dehydrogenase 3; EDA: ectodysplasin A; EGF: epidermal growth factor; FGF: fibroblast growth factor; IGF: insulin growth factor; IL: interleukin; SHH: sonic hedgehog; SCF: stem cell factor.

Secretome components	References
ALDH3 activator	[24]
EDA	[25]
EGF	[26]
FGF2	[27]
FGF7	[28, 29]
FGF10	[29–31]
Heparan sulfate	[31, 32]
IGF1	[33]
IL-6	[34]
SHH	[35]
SCF	[32]
Wnt	[36–38]

**Table 2:** In vivo and in vitro tested oral stem cell lines for salivary gland regeneration. SG: salivary gland, BM: bone marrow, MSC: mesenchymal stem cells, ESC: embryonic stem cells, and iPSC: induced-pluripotent stem cells.

Tested cell sources	Origin (species)	References
Major SG progenitor/stem cells	Mouse, rat, human	[46–48]
Minor SG epithelial cells	Human	[49]
BM-derived stem cells	Human	[50, 51]
BM-derived MSC	Human	[14, 52]
Adipose-derived MSC	Human	[53, 54]
Minor SG-derived MSC-like cells	Human	[55]
Amniotic epithelial cells	Human	[56, 57]
ESC	Mouse	[58]
iPSC	Mouse	[59]

**Table 3:** List of secretome components (cytokines, growth factors, and proteinases) from adult stem cells (e.g., bone marrow-derived stem cells and adipose mesenchymal stem cells) that can be potentially used in SG regeneration strategies. More details about each secretome component can be found in [18, 23]. FGF: fibroblast growth factor; FLT3: Fms related tyrosine kinase 3; G-CSF: granulocyte-colony stimulating factor; GM-CSF: granulocyte macrophage-colony stimulating factor; HGF: hepatocyte growth factor; IGF: insulin growth factor; IL: interleukin; MMP: matrix metalloproteinase; OPN: osteopontin; PF4: platelet factor 4; SCF: stem cell factor; SDF1: stromal cell derived factor-1; VEGF: vascular endothelial growth factor.

Secretome components	References
CD26	[16]
FGF1	[16]
FLT-3	[62]
G-CSF	[62]
GM-CSF	[17]
HGF	[16]
IGF-1	[17]
IL-1ra	[16]
IL-6	[17]
IL-16	[16]
MMP8	[16]
MMP9	[16]
OPN	[16]
PF4	[16]
SCF	[62]
SDF1	[16]
VEGF	[17]
